# Toward a Flexible and Efficient TiO_2_ Photocatalyst Immobilized on a Titanium Foil

**DOI:** 10.1021/acsomega.1c02862

**Published:** 2021-08-25

**Authors:** Živa Marinko, Luka Suhadolnik, Barbara Šetina Batič, Vid Simon Šelih, Boris Majaron, Janez Kovač, Miran Čeh

**Affiliations:** † Department for Nanostructured Materials, Jožef Stefan Institute, Jamova 39, 1000 Ljubljana, Slovenia; ‡ Jozef Stefan International Postgraduate School, Jamova 39, 1000 Ljubljana, Slovenia; § Vacuum Science and Optoelectronics, Institute of Metals and Technology, Lepi pot 11, 1000 Ljubljana, Slovenia; ∥ Center for Validation Tehnologies and Analytics & Department of Analytical Chemistry, National Institute of Chemistry, Hajdrihova 19, 1001 Ljubljana, Slovenia; ⊥ Department of Complex Matter, Jožef Stefan Institute, Jamova 39, 1000 Ljubljana, Slovenia; # Faculty of Mathematics and Physics, University of Ljubljana, Jadranska 19, 1000 Ljubljana, Slovenia; ∇ Department of Surface Engineering, Jožef Stefan Institute, Jamova 39, 1000 Ljubljana, Slovenia

## Abstract

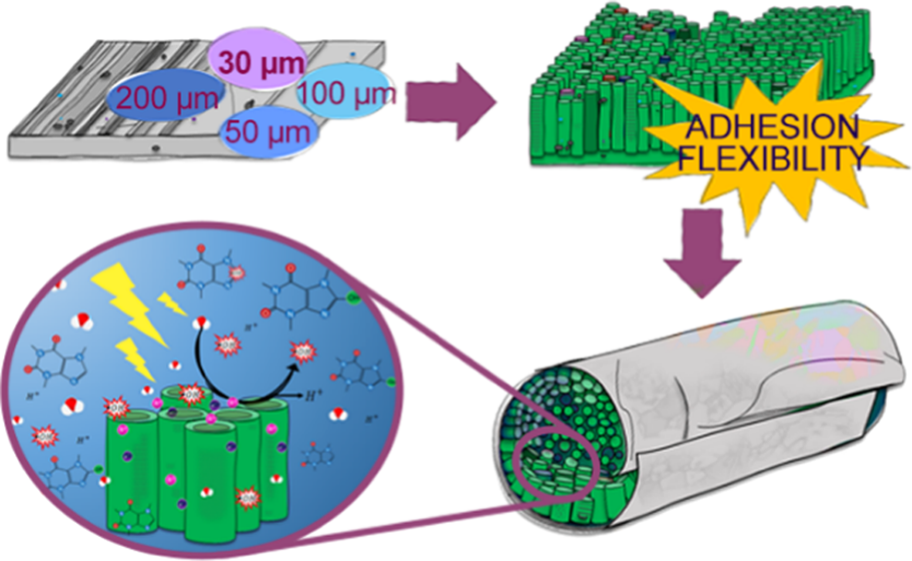

Titanium foils of different thicknesses were anodized, and the photocatalytic activity of the resulting TiO_2_ nanotube (NT) layers was determined. All of the titanium foils were anodized simultaneously under identical experimental conditions to avoid the influence of the aging of the anodizing electrolyte and other anodization parameters, such as voltage, time, and temperature. To characterize the microstructures of the titanium foils, we used electron backscatter diffraction (EBSD), scanning electron microscopy (SEM), and stylus profilometry analyses. The adhesion was tested with a Scotch tape test and the morphology of the TiO_2_ NTs was studied in detail using the SEM technique, while the surface areas of the TiO_2_ NTs were determined using a three-dimensional (3D) optical interference profilometer. With X-ray diffraction (XRD) and X-ray photoelectron spectroscopy (XPS), the chemical composition and structure of TiO_2_ oxide were established. The degradation of caffeine under UV irradiation was measured with a high-precision UV–vis–IR spectrophotometer, and the photoluminescence method was used to confirm the photocatalytic behavior of the TiO_2_ NT layers. The influence of the intrinsic properties, including twinning and the grain boundaries of the starting titanium foils with similar chemical compositions, was determined and explained. Finally, we identified the main characteristics that define a highly effective and flexible photocatalyst.

## Introduction

Increasing levels of water pollution demand improved technologies for the degradation of organic pollutants in wastewaters. The photocatalytic degradation of such organic pollutants using a TiO_2_ catalyst is one of the most attractive technologies to deal with this problem, as recognized by Fujishima and Honda^1^ and in the pioneering work of Frank and Bard.^2^ Since then, many research groups have contributed to this topic because of its immense potential and the possibility of practical use.

The technological application of TiO_2_-based photocatalytic degradation of organic compounds using photocatalysis requires not only a high photocatalytic activity and a large surface area of the catalyst but also strong adhesion to a substrate or scaffold.^3−5^ Flexibility of the active catalyst would also be beneficial in many applications.^6−9^

TiO_2_ nanotube (NT) layers can be grown on titanium metal with the electrochemical process of anodic oxidation.^10−18^ As a result of the growth mechanism, such TiO_2_ NTs exhibit excellent adhesion to the titanium substrate. For this reason, they can be used directly as a platform for the degradation of organic pollutants in wastewaters.^19^ Moreover, due to their connection with the substrate, they will not be released into the environment during the degradation process. Due to the many factors that influence TiO_2_ NT growth, a lot of research has been done on the anodic oxidation of metal titanium in various forms. TiO_2_ NT growth and photocatalytic activity are tuned with the parameters of the anodization process, like voltage, time, electrolyte composition, and pH. Additionally, the TiO_2_ NTs can be modified with post-treatment processes, such as annealing or doping to move the band gap closer to visible light.^12,20−24^ However, much less work has been done on the influence of the titanium substrate’s properties on TiO_2_ NT growth. While some earlier research was focused on the crystallographic orientation of the grains in the titanium foil and the morphology of the starting surface,^25−29^ information on how the thickness of the foil influences the TiO_2_ NT growth during anodization is scarce.^30^

Titanium foils, like any other metal foil, can be produced using cold or hot rolling. The titanium foils in this study were produced with cold rolling. A so-called roll coating can form during the process, which greatly deforms the surface of titanium.^31^ Moreover, the transfer of the material between the titanium sheets and the rolls often results in surface contamination and increased surface roughness.^32^ However, the largest impact on the microstructure of titanium is the occurrence of plastic deformations, such as dislocation slips and twinning.^33−37^ Chun et al.^35^ studied twinning in commercial titanium foils and the formation of twins during the cold rolling process. The twinning was activated at a lower titanium thickness reduction, while during higher deformations, dislocation slip was the only mechanism of deformation. They also confirmed the formation of compressive twinning {11–22} <11-2-3> and tensile twinning {10–12}<10-1-1> and observed the formation of secondary and tertiary twins during the rolling process.

Of the many processes used to synthesize photoactive TiO_2_ NTs, only anodic oxidation results in ordered layers of rigidly attached TiO_2_ NTs on a metal titanium substrate. Any form of titanium can be anodized, meaning such catalysts can be used in a wide range of applications. We anodized titanium foils from the same supplier, but with different thicknesses. By combining various analytical techniques, we studied the influence of the chemical, structural, and morphological properties of the starting titanium foils on the growth mechanism, NT morphology, and the resulting photocatalytic activity of the anatase TiO_2_ NTs. The main goal of our investigation was to synthesize a flexible photocatalyst that would withstand the stress and deformation of the otherwise flat TiO_2_ NT layer. We successfully determined which foil thickness results in the most rigidly attached TiO_2_ NT layer during the anodization of a flat titanium foil.

## Results and Discussion

### Microstructure Properties of the Starting Titanium Foils

**Figure 1:**
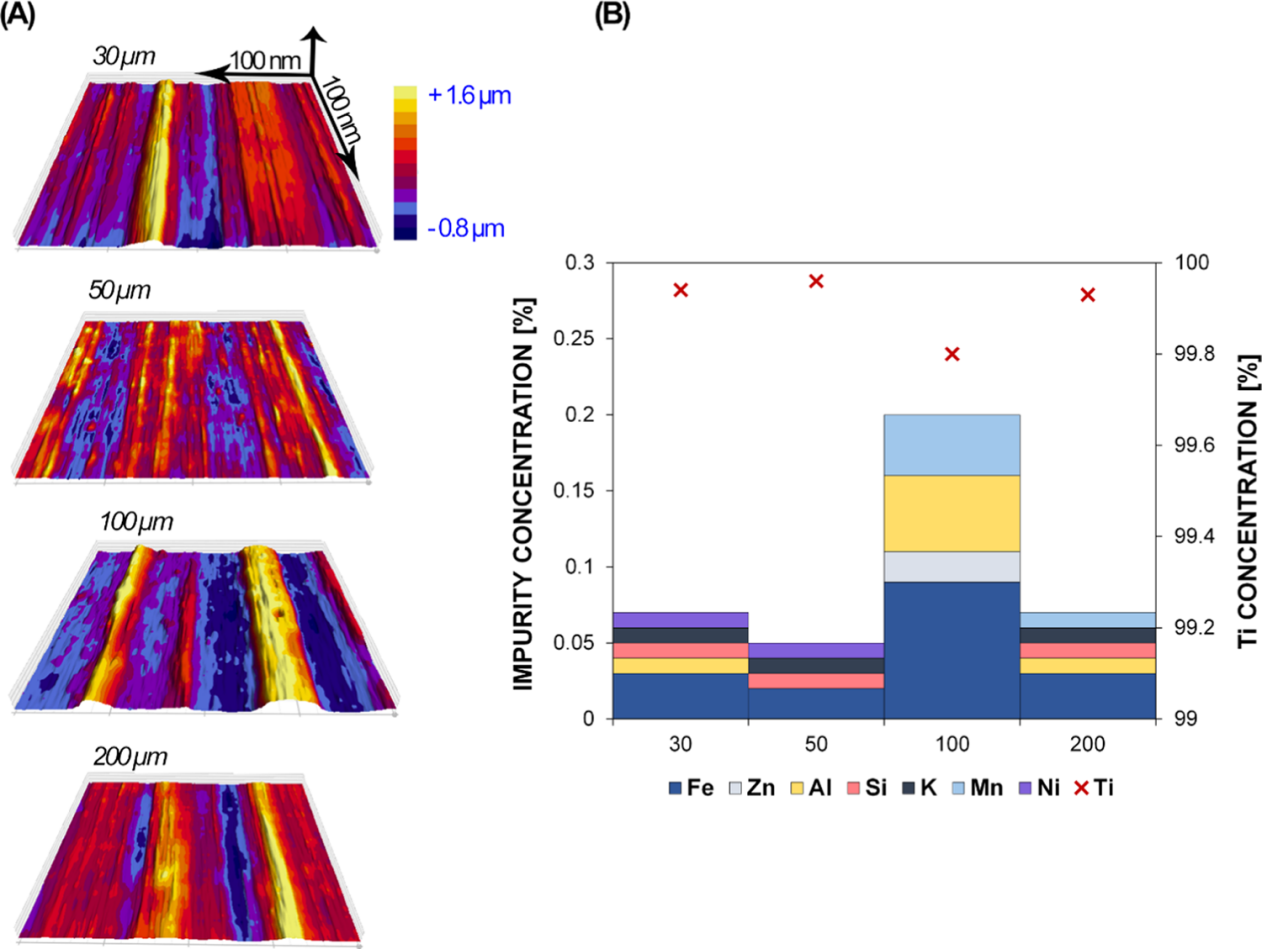
(A) 3D stylus profilometry mapping of the four metal titanium foils with different thicknesses. The marked *z*-axis is equal for all samples. (B) Inductively coupled plasma optical emission spectrometry (ICP-OES) analyses of all four foils. The purity of the titanium foil is marked with an “x” and the impurities indicated with color bars.

The surface morphology investigation of the as-received titanium foils using a field emission gun scanning electron microscope (FEG-SEM) revealed significant differences between the samples of different thicknesses (30, 50, 100, and 200 μm). Different rolling patterns, scratches, and other markings were the most significant features, as shown in Figure S1. It can be seen that the 30 and 100 μm samples have a similar surface with more obvious surface variations than the other two samples, where the difference in surface roughness is smaller. Meanwhile, the 200 μm sample appeared with pores on the surface and the 50 μm sample with the least surface undulations among the samples. A strong relationship between the surface roughness and the TiO_2_ NT growth has been reported previously by many research groups.^38−40^ To better understand the scale of the surface undulations, we three-dimensionally (3D) mapped each sample with a stylus profilometer. The results revealed the anisotropic nature of the foils’ surfaces. The calculated average roughness factor (*R*_a_) and the peak-to-valley factor (*R*_t_), assessed over a 4 mm line across the surface, are presented in Table 1, and the roughness profiles are shown in Figure 1A. The highest surface roughness and the average peak deviation were observed for the 100 μm sample. The thinnest foil (30 μm) has a similar morphology but lower values of both factors. In contrast, the 200 μm sample has an even distribution of hills and valleys along the rolling direction, and the 50 μm sample exhibits the flattest surface among the tested foils with the lowest average roughness values. Since the thinnest titanium foil was produced by cold rolling the thickest titanium foil, we expected that both roughness factors would have the highest values in the 200 μm sample. However, significant undulations, in the form of individual ripples, from 0.8 μm in depth to 1.6 μm in height, were characteristic for the 100 μm sample. This resulted in higher roughness compared to the 30, 50, and 100 μm samples, where the rolling pattern (the distribution of peaks and valleys) was even and the *z*-axis values were significantly lower.

**Table 1:** Average Roughness (*R*_a_) and Peak-to-Valley Factor (*R*_t_) Measurements

foil thickness (μm)	*R*_a_ (μm)	*R*_t_ (μm)
30	0.14 ± 0.01	1.23 ± 0.16
50	0.11 ± 0.02	1.42 ± 0.42
100	0.29 ± 0.01	2.52 ± 0.33
200	0.16 ± 0.01	1.24 ± 0.12

The chemical composition of all four samples showed that they contained more than 99.8% titanium (Figure 1B). The least pure was the 100 μm sample, with 99.8% of titanium in the foil. The main impurity detected in the titanium foil was iron.^40^ The values were decreasing from 100, 200, and 30 to 50 μm sample with percentages of 0.09, 0.03 (200 and 30 μm), and 0.02%, respectively. Another chemical element that had a significantly higher concentration in the 100 μm sample was aluminum, with 0.05%. Other impurities identified in all four samples were zinc, silicon, manganese, nickel, and potassium. Although the measured concentrations are low, the impurities could influence the physicochemical properties and the NT growth through incorporation in the anodic oxide. A similar process is known for the migration of fluoride ions from the electrolyte to the oxide layer, and their influence on the NT formation and the related photocatalytic activity.^41^ Moreover, higher impurity concentrations could result in surface functionalization or modulation of the photocatalytic properties of the TiO_2_ NTs, as can be seen in the anodization of Ti alloys.^42^

**Figure 2:**
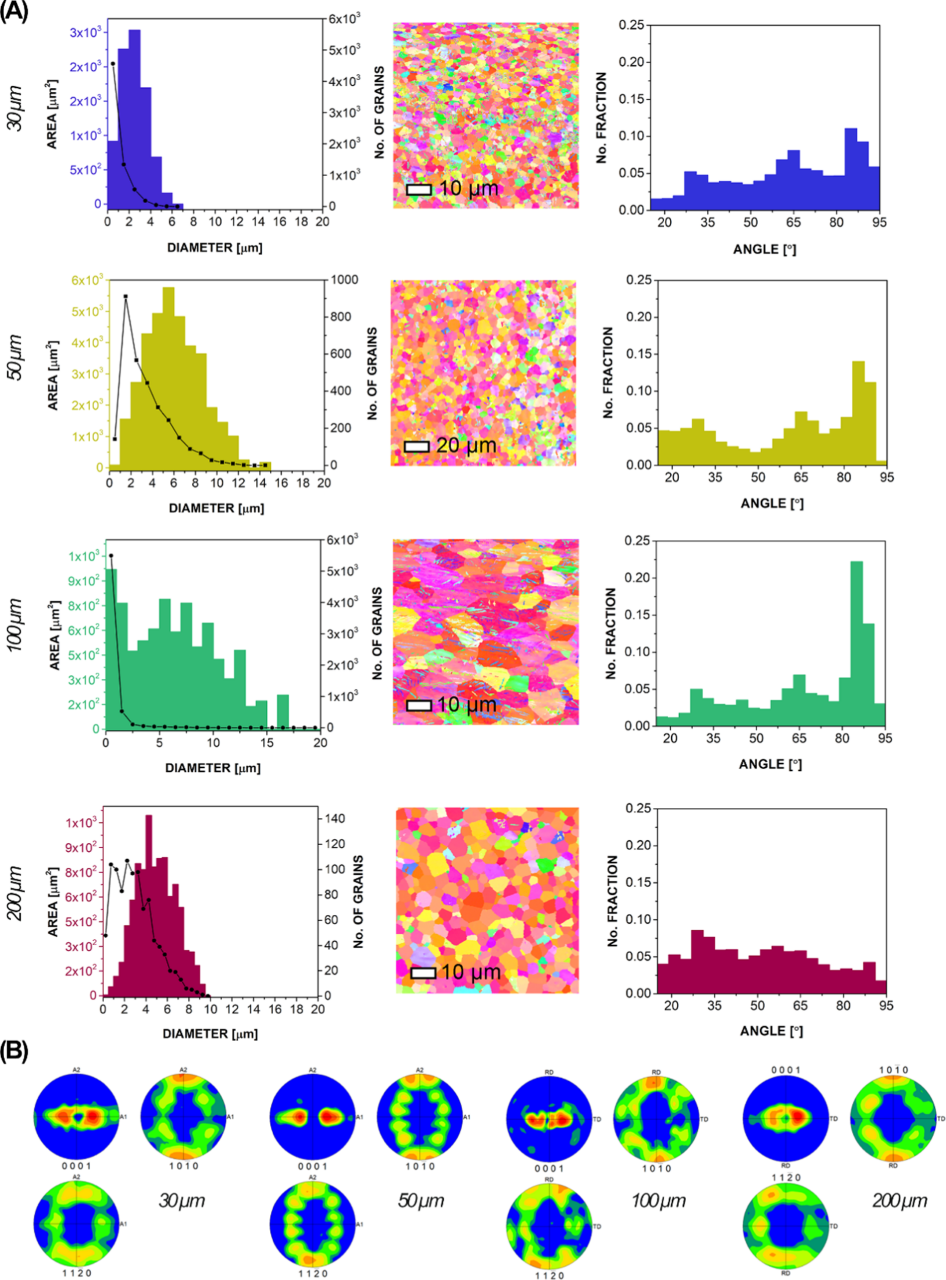
(A) Orientation maps (IPF-Z color coded) for titanium foils of different thicknesses are shown between two histograms. The grain size distribution on the left, where the bars represent the area of the grains and the line shows the number of grains, and the grain boundary misorientation angle distribution histograms on the right. (B) The corresponding pole figure plots of grain orientations obtained from the EBSD data.

The crystallographic orientation map of individual grains in the foils for each sample is shown in the center columns of Figure 2A. To the left of the maps are the corresponding grain size histograms, and on the right are the corresponding histograms of the grain misorientation angles between 15 and 95°. Pole figure plots obtained from the EBSD are shown in Figure 2B. Additionally, refer to Figure S2 for the inverse pole figures (IPF) for all samples. Titanium crystallizes in the hexagonal close-packed crystal system. Backscatter imaging and EBSD analyses showed only the presence of α-Ti. This system has a low lattice symmetry and few slip systems, so the texture forms easily during deformation or processing, such as rolling and annealing. The principal slip systems in Ti are prismatic {10–10} <11–20>, secondary basal {0001} <11–10>, and two pyramidal {10–11} <11–20> and {11–22} <11–23>. During the cold rolling, titanium tends to form textures with basal poles tilted at ±20 to 40° from the normal direction.^43^ The occurrence of twinning depends on the surface deformation of the starting titanium foil. All of the analyzed titanium foils exhibited a strong rotated basal texture with a distinct TD split associated with rolling.^34^ However, the twinning was reduced when the titanium foil was thinner.^44^ The rotation was between 25 and 40°. A very strong texture associated with tensile twinning ({10–12} <−1011>) was observed in the 100 μm sample, where twins were also prominent in the microstructure. These results provide important insights into the evolution of the texture of the thinnest titanium foil.

Cold rolling additionally influences the grain size since more pronounced rolling results in smaller grains. The size of the grains can be directly linked to the material’s strength; the smaller the grains, the more strength the material has.^45^ The average grain size was determined for each titanium foil thickness and the grain distribution appeared specific for each sample. A Gaussian distribution of grains was specific for the 30 μm thick foil with grains averaging 2.5 μm in diameter, for the 50 μm foil with grains averaging 5.5 μm in diameter, and for the 200 μm foil with an average grain size of 4.25 μm in diameter. A significantly wider and more uniform distribution was characteristic for the 100 μm thick foil with grain diameters up to 10 μm. Interestingly, with the decreasing thickness of the titanium foil, the grains were not elongated but instead remained more or less equiaxed.^37^ However, some elongation was observed due to the occurrence of slip.^35^ The distribution of the grains was also observed in the polished titanium foil’s cross sections (not shown here) under an optical microscope. Although the grains were evenly distributed over the foils’ cross section, a notable feature was observed for the 100 μm foil. While the 30, 50, and 200 μm foils were characterized by small grains, the 100 μm sample was dominated by significantly larger grains (a smaller number of grain boundaries), which could influence the material’s ductility due to reduced residual stress at the grain boundaries.

### Characteristics of the Annealed TiO_2_ NT Layers

**Figure 3:**
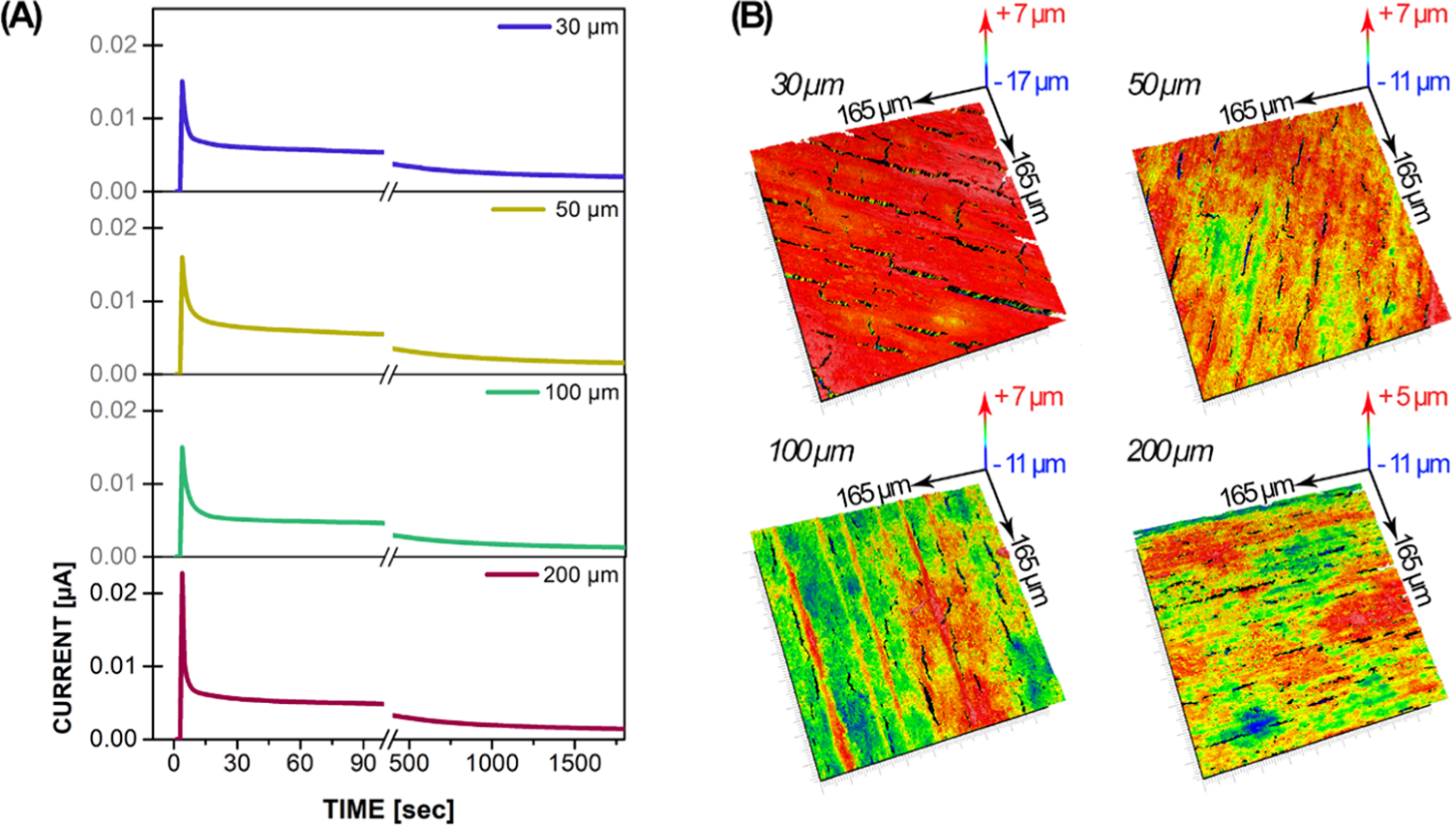
(A) Current/time measurements during the anodization of titanium foils with different thicknesses in the fresh electrolyte. (B) TiO_2_ NTs’ surface roughness as measured with a 3D interference optical profilometer.

The behavior of the anodization current was observed for the different thicknesses of the titanium foils. During the process, amorphous TiO*_x_* was grown. The anodization current/time curves measured during the first 30 min of anodization with a fresh electrolyte for all foils are presented in Figure 3A. After that time, the current oscillations were not significant as the growth reached a steady state. At the beginning of the anodization of the 200 μm foil, the current was the highest, i.e., 0.023 μA, whereas, for the 100, 50, and 30 μm foils, the currents were 0.015, 0.016, and 0.015 μA, respectively. After the initial stage of anodization, the current gradually decreased with time, until it reached a steady-state value of 0.001–0.002 μA.

Figure 3B shows the TiO_2_ NT layers’ surfaces, measured with a 3D interference optical profilometer to determine average surface roughness. Surface regions that are higher (red) or lower (blue) than the average surface height (green) are presented. The measurements show that the thinnest foil (30 μm) exhibits the most prominent average surface roughness (0.5 μm) and average peak-to-valley (21.3 μm) profile compared to the other three samples. Analyses of the surface profile maps revealed that the TiO_2_ NT layer cracks are also significantly wider than those in the other samples. Moreover, individual NTs inside the cracks were visible in the cross sections. This contributed a great deal to the larger surface area since the overall measured surface roughness is the sum of the TiO_2_ NT surface and the NT wall surfaces exposed in the cracks. The second highest average surface roughness (0.4 μm) and average peak-to-valley (15.2 μm) profile were measured for the 100 μm thick anodized foil. Analyses with a stylus profilometer (see Figure 1A) showed that the key contributions to the roughness were the individual surface deviations across the NT layer (red lines in Figure 3B). Compared to the other three samples, there are not many cracks in the TiO_2_ NT layer in the 100 μm sample. The anodized 200 and 50 μm thick foils have the lowest values, i.e., 0.3 and 0.2 μm, for the average surface roughness, and 13.1 and 12.1 μm, for the average peak-to-valley profile, respectively.

While the morphological characterization of the annealed TiO_2_ NTs resulted in interesting findings, their crystallographic structures and chemical compositions did not show significant differences (see Section S2 in the Supporting Information). Analyses of the micrographs presented in Table 2 show that the NTs’ average length is approximately 21 μm, except for the 100 μm sample, where the NTs are shorter (18.0 ± 0.9 μm). Analyses of the detached TiO_2_ NT layer’s bottom part revealed the double-walled NTs. The average thickness of the outer wall is similar for all of the samples, on average 18 nm. Meanwhile, differences in the inner wall thicknesses were more significant, but still within the measurement uncertainty. Variations in the measured values suggest that the TiO_2_ NTs were not uniform. The overall diameter of the NTs (including the hollow part) was similar across the four samples (from 145 to 151 nm) and the analyses of the top surface of the TiO_2_ NTs revealed that the double wall at the bottom was converted into single-walled NTs at the top during the growth/crystallization process.^46^ The NTs near the top surface were, on average, 40 nm narrower in diameter than those at the bottom, which could be attributed to the prolonged etching by fluoride ions during anodization or removal of the carbon remnants on the inner wall during the annealing process.^47,48^

**Table 2:** Analyses of TiO_2_ NTs’ Dimensions

		double-walled NTs			single-walled NTs
foil thickness (μm)	NT length (μm)	outer wall (nm)	inner wall (nm)	NT diameter (nm)	NT diameter (nm)
30	21.6 ± 0.7	18.6 ± 2.8	33.9 ± 4.1	148 ± 5	101 ± 16
50	21.9 ± 0.4	17.6 ± 2.7	32.7 ± 3.8	150 ± 11	94 ± 9
100	18.0 ± 0.9	18.1 ± 2.5	37.4 ± 5.2	151 ± 13	104 ± 8
200	21.2 ± 1.5	17.9 ± 2.5	36.3 ± 4.3	146 ± 9	97 ± 10

To investigate the adhesion of the TiO_2_ NTs, we performed the Scotch tape test.^49,50^ Due to the easy handling and no loss of material on the thinnest sample, it was expected that the adhesion would be the strongest, with the NT layer on the thickest foil having the poorest adhesion. However, by observing the area around the X-cut under the optical and FEG-SE microscopes, we found that almost all of the NTs peeled off the surface of the 30 μm foil easily (Figure S6). In contrast, the NTs’ adhesion was the strongest to the 100 μm foil, followed by the 50 and 200 μm foils. The results are shown in Figure S6 in the Supporting Information. The adhesive properties of the NT layer are closely related to (1) the NT length, (2) the number of cracks in the TiO_2_ NT layers, and (3) the surface roughness of the NTs.^51−53^ Longer NTs and a larger number of cracks in the NT layer tend to weaken the adhesion. Meanwhile, uniform TiO_2_ NT layers tend to strengthen it. Cao et al.^51^ suggested that interfacial adhesion between the NT layer and the titanium foil is responsible for the adhesive strength, which decreases with the increase of the thickness of the TiO_2_ layer. To conclude, we observed that the reason for the delamination and detachment of the TiO_2_ layer is not straightforward; instead, it is a complex phenomenon influenced by the above properties.

### Measurements of the Photocatalytic Degradation

**Figure 4:**
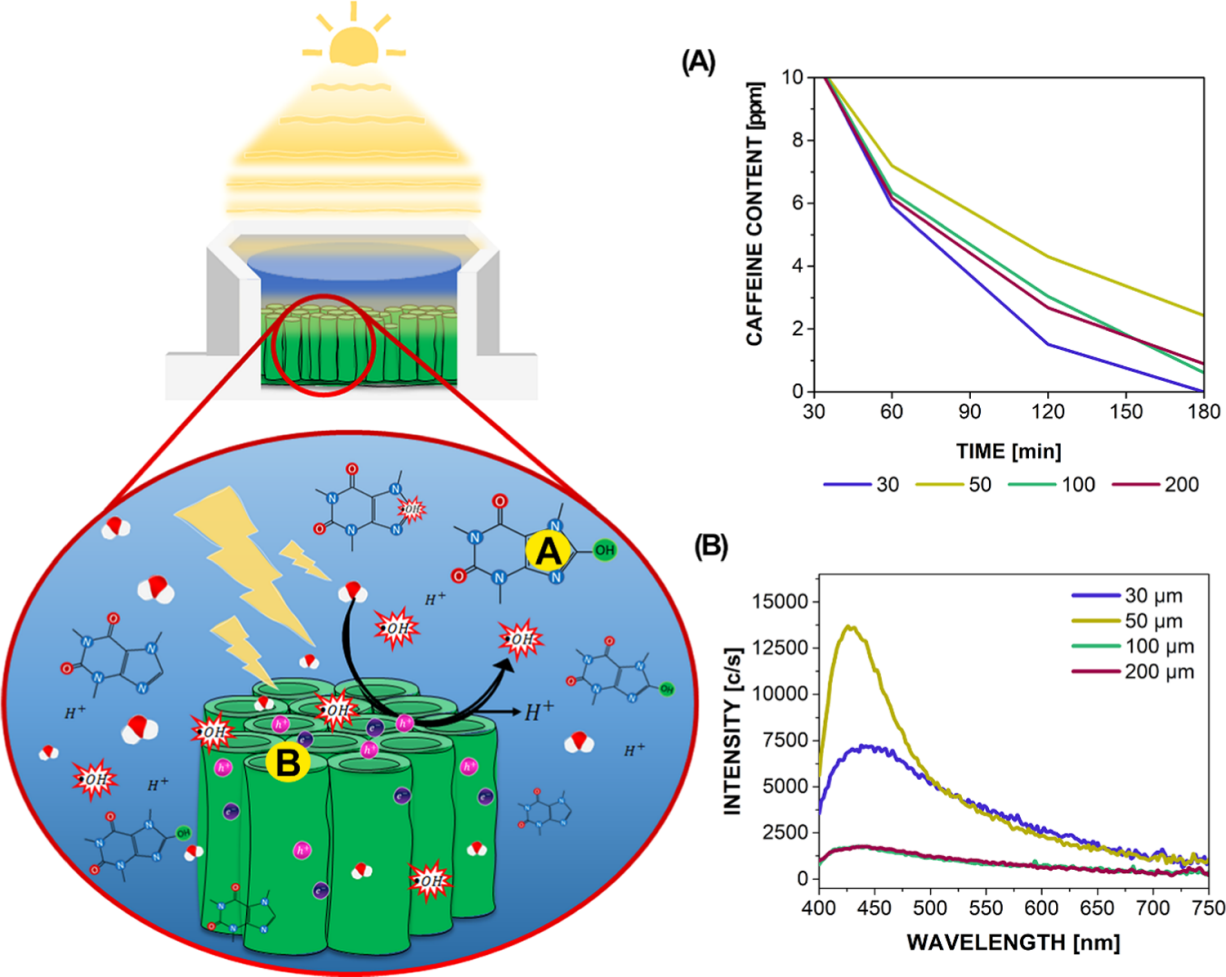
Photocatalytic activity of four TiO_2_ NTs samples. Main aspects of this section’s research are presented. (A) Caffeine degradation and (B) photoluminescence measurements.

The schematic presentation of our results related to photocatalysis and the photocatalytic activities of the as-synthesized TiO_2_ NTs is presented in Figure 4 and is linked to the caffeine degradation results (Figure 4A) and the photoluminescence (PL) measurements (Figure 4B). Both are closely connected by providing information on some level of the photocatalytic reaction.

The caffeine degradation test, presented in Figure 4A, showed that all of the samples have the ability to degrade molecules in a 10 ppm initial aqueous solution within 3 h of UV exposure (additionally, refer to Figure S7 for the exponential curve fitting for all of the samples). The drop in caffeine concentration occurred at different rates. The corresponding initial rate constants were calculated by the following formula, assuming a first-order reaction^54^
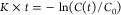
where *C* is the caffeine concentration at time *t* and *C*_0_ is the initial caffeine concentration.^55^ The results in Table 3 revealed that the 30 μm sample exhibited the best photocatalytic activity by far, followed by the 100 and 200 μm samples, while the 50 μm sample showed the least activity. The 30 μm sample achieved the complete degradation of caffeine in less than 3 h, followed by the 100 μm sample with 99.4%, the 200 μm sample with 99.1%, and the 50 μm sample with 97.6% degradation efficiency. These results confirmed that the 30 μm foil gave the best photocatalytic result since it has the largest specific surface area (Figure 3B), which is the primary factor for degradation efficiency.^56^

**Table 3:** Reaction Rate Constants for Caffeine Degradation

foil thickness (μm)	reaction rate constant *k* (×10^–2^ min^–1^)
30	2.12 ± 0.13
50	1.04 ± 0.06
100	1.59 ± 0.12
200	1.65 ± 0.07

Finally, the photoluminescence (PL) signal is attributed to electron–hole recombination, meaning that fewer recombinations result in a lower PL signal. These measurements provided us with additional knowledge about the presence of the TiO_2_ photoactive sites. The measured PL spectra of the TiO_2_ NT layers are presented in Figure 4B (lines are calculated from multiple scans). The 50 μm foil had a narrower and high-intensity peak around 430 nm. This measurement points to poor photocatalytic activity, which was already confirmed with the worst caffeine degradation measurements among the four samples. In addition, all samples possess wider bands between 400 and 540 nm. Broader photoluminescence spectra are typical for the presence of oxygen vacancies and defects on the TiO_2_ surface.^57,58^ With oxygen vacancies, the photoexcitation shifts slightly toward visible light, enhancing the photocatalytic activity.^59^ Meanwhile, the lowest measured intensities were for the 100 and 200 μm foils, which indicated higher photocatalytic activity due to a decreased recombination rate and increased amounts of adsorbed O_2_. The 50 and 200 μm samples showed a significant drop in photostability during the PL measurements. The PL measurements confirmed the influence of defect sites responsible for the signal, i.e., from the impurities and defect sites that originate from the titanium foil and can be found on the TiO_2_ NT layer (Figures 1, 2, and S5) to oxygen vacancies and titanium species like Ti^3+^ in anatase TiO_2_ (Figure S3). In particular, the defect sites associated with the oxygen vacancies are preferred for the adsorption of molecular oxygen onto the TiO_2_ surface.^60^ Please refer to Characteristics of the Annealed TiO_2_ NT Layers section in the Supporting Information for more information regarding X-ray photoelectron spectroscopy (XPS) and TiO_2_ NT’s top surface analysis.

## Conclusions

This paper presents thorough structural, morphological, and photocatalytic degradation studies of rigidly attached TiO_2_ NT layers obtained by the anodic oxidation of titanium foils for use in flexible annular photocatalytic reactors for the degradation of organic pollutants in wastewaters. We investigated titanium foils with four different thicknesses. While many studies have noted the importance of the surface properties of the starting titanium material on the structural and morphological TiO_2_ NT characteristics, only very few studies focused on a correlation with the TiO_2_ NTs’ photocatalytic activity.

Significant differences were observed in the surface roughness, chemical composition, grain size distribution, and crystallographic orientation after the stylus profilometry, ICP-OES, and EBSD analyses were performed. It was shown that even minor differences in the titanium foil’s purity could greatly improve/deteriorate the structural, morphological, and optical properties. Moreover, a titanium substrate with higher purity results in a TiO_2_ NT layer with higher photocatalytic activity.^61^ The least pure sample (100 μm) with a significant iron content resulted in the second best active sample. However, the chemical composition is not the only determining factor in photocatalytically active NTs. Our study confirmed the influence of the anodization parameters and the titanium properties on the formation of the passive oxide layer and the NT formation during the process of growth. It is known that ultrafine-grained metals contain more surface defects and have a higher density of grain boundaries, which means a larger number of nucleation sites for the growth of NTs. Consequently, they are less resistant to corrosion than substrates with larger grains during the anodization process, leading to faster NT growth and longer NTs. This effect was clearly seen in our experiments, where the 100 μm foil sample had the largest grains and therefore resulted in the shortest TiO_2_ NTs.

Moreover, the morphological and compositional changes of the NT layer were successfully determined with various characterization techniques. Photocatalysis is a complicated process that involves morphological and optical properties. Significant morphological differences reflected in the photocatalytic activity were confirmed by measuring the optical properties and the efficiency of the caffeine degradation. Measurements of surface roughness showed that the highest surface area was available on the 30 μm foil, where complete degradation of caffeine was also measured. A considerable number and width of cracks were observed in this sample. These affect the light scattering during the production of radicals and, with that, reduce the concentrations of radicals produced. The 100 μm foil sample, which exhibits the least cracked TiO_2_ NT layer surface also had the most adhesive NTs with respect to the substrate (measured with the Scotch tape test). Meanwhile, the adhesion test of TiO_2_ NTs showed how poorly the TiO_2_ NTs from the thinnest foil were attached to titanium. Our study showed that the main factors contributing to strong adhesion were the NT surface with fewer cracks and shorter NTs, which were the main characteristics of the 100 μm foil sample.

Our results showed that the thinnest titanium foil, although with the best caffeine degradation result, is not the most appropriate for preparing a flexible photocatalyst due to the poor adhesion of NTs to the titanium substrate. On the contrary, the 100 μm sample showed the best results. This sample showed high photocatalytic activity and the ability to bend without the risk of cracking the NTs or detaching them from the substrate.

## Experimental Methods

### Characterization of the Titanium Foil

Titanium foils (99.9% purity, Baoji Lyne Metals) were processed from a titanium slab with cold rolling. We used titanium foils with starting thicknesses of 30, 50, 100, and 200 μm. The surface morphologies of the as-received titanium foils were examined using a field emission gun scanning electron microscope (FEG-SEM; JSM-7600F, JEOL), while the surface roughness, measured in a direction perpendicular to the factory rolling, was determined using a stylus profilometer (2 μm tip; DektakXT, Bruker).

The texture of the titanium foils was assessed by electron backscatter diffraction (EBSD; Hikari Super, EDAX). Before the EBSD analyses, the titanium foils were finely polished with 3 μm diamond paste, and additional OP-S (colloidal silica) polishing was applied for 5 min. Data postprocessing and analyses were performed using the OIM software package (EDAX).

The chemical composition of the titanium foils was determined by inductively coupled plasma optical emission spectrometry (ICP-OES) after the foils were digested, as follows. Accurately weighed (100 mg) pieces of the foils were digested on a hot plate using concentrated hydrochloric acid. Five milliliters of HCl (Suprapur, Merck) was added into the Ti foils and slowly heated in a covered beaker for 5 h, before being left to cool overnight. As titanium strongly resists acid attack, this procedure had to be repeated three times over several days before the Ti foils completely dissolved. The digestates were then diluted to 50 mL and measured by ICP-OES (Varian 715-ES) in a semiquantitative mode, thus revealing the chemical composition of the Ti foils.

### Synthesis of TiO_2_ NT Layers by Anodic Oxidation

The titanium foils were cut into 15 × 15 mm^2^ samples and ultrasonically cleaned with acetone for 10 min. The samples were then rinsed with absolute ethanol and deionized water and then dried under a stream of nitrogen. The anodization electrolyte was a mixture of ethylene glycol (99.99%, Carlo Erba) and a solution of 0.3 wt % NH_4_F (Sigma-Aldrich) in 2 vol % deionized water. Anodic oxidation was performed in a two-electrode electrochemical cell connected to a constant 60 V DC power supply for 3 h. A data logger (Agilent) monitored the electrical current during the entire process. The grown amorphous TiO_2_ NTs on the titanium foils were rinsed with ethanol and dried under a stream of nitrogen. Afterward, these amorphous TiO_2_ NTs were transformed into crystalline TiO_2_ NTs with the anatase structure by annealing in a muffle furnace (Nabertherm) at 450 °C for 1 h, with heating and cooling rates of 5 °C/min.

### Measurements of the Photocatalytic Degradation

The photocatalytic activity of the anodized TiO_2_ NTs was measured with the degradation of a model compound, i.e., caffeine, due to its presence in wastewaters and its easy and safe handling.^62,63^ Titanium foils with an anodized area of 0.8 ± 0.03 cm^2^ were put in a Petri dish with 5 mL of an aqueous 10 ppm caffeine solution and placed in a sterilizer (Kambič I-265 CK UV). Under constant stirring, 200 μL of the solution was collected after 30 min in the dark. Afterward, the samples were illuminated with UV light (Ultra-Vitalux, OSRAM, UVA—from 315 to 400 nm and UVB—from 280 to 315 nm) and collected at 60, 120, and 180 min for analyses using a high-precision UV–vis–IR spectrophotometer (Lambda 950, PerkinElmer).

### Characterization of the TiO_2_ NT Layer

The morphologies of the anodized and annealed TiO_2_ NT layers were characterized using a FEG-SEM. The average surface roughness of the TiO_2_ NT layers was determined and evaluated over the surface with a 3D interference optical profilometer (ZeGage ProHR with MX Software Package, Ametek Zygo). After annealing, X-ray diffraction (XRD; X’Pert PRO, Panalytical) analysis was used to determine the crystallinity and crystal structure of TiO_2_ NTs. The samples were scanned using Cu Kα radiation in a 2θ range of 20–80° for 100 s over a 5 mm mask. Peaks were identified with the X’Pert HighScore Plus program using the International Centre for Diffraction Data (ICDD) PDF-4+2019 database.

The photoluminescence (PL) properties of the TiO_2_ NT layers were assessed using a spectrofluorometer (QuantaMaster 8000, Horiba-PTI) with a low-noise photomultiplier (Hamamatsu R2658). All of the samples were excited at 370 nm, and the emission spectra were measured between 400 and 750 nm. X-ray photoelectron spectroscopy (XPS) was used to analyze the upper 5 nm of the TiO_2_ NT layers on a surface area with a diameter of 0.4 mm. The analyses were performed using a PHI-TFA XPS spectrometer (Physical Electronics Inc., Eden Prairie) with an Al monochromatic X-ray source.

Finally, the adhesion of the rigidly attached TiO_2_ NTs was estimated with the Scotch tape test, following the ASTM D3359 standard. Two cuts, approximately 10 mm long and at an angle of 30–45° (X shape), were made into the TiO_2_ NT layer using a clean steel blade. Just enough pressure was applied so that the blade tip penetrated the NT layer and exposed the Ti substrate. Next, a piece of Scotch tape was placed on the cut, pointing in the same direction as the smaller angles. It was pressed gently to ensure good contact with the TiO_2_ layer. After the application, the tape was removed with a single quick pull. Afterward, the foils and the Scotch tape were observed under an optical microscope (Discovery V8, Carl Zeiss Microscopy). Several micrographs were stitched together and analyzed in the ImageJ program (1.53e, NIH) to determine the loss of TiO_2_ NTs around the cut. Larger detached and/or delaminated areas distant from the cut were not included in the analysis. Following the analysis under the optical microscope, morphological changes on the titanium substrate and TiO_2_ NT layers that occurred after the tape was pulled were inspected using the FEG-SEM (JSM-7600F, JEOL).

## Supporting Information Available

SEM images of the metal titanium foil of different thicknesses (Figure S1); inverse pole figures (IPFs) for all samples (Figure S2); surface chemical composition in at. % and the O/Ti ratio for all samples (Table S1); high-resolution XPS spectra for (a) Ti 2p, (b) O 1s, and (c) C 1s peaks for all TiO_2_ NT samples (Figure S3); XRD measurements for each sample (Figure S4); FEG-SEM micrographs of TiO_2_ NTs for each foil thickness (Figure S5); adhesion of TiO_2_ NTs performed with the Scotch tape test on (a) 30 μm, (b) 50 μm, (c) 100 μm, and (d) 200 μm samples (Figure S6); photocatalytic degradation of caffeine (dots) for all four samples; and exponential curve fitting (lines) for 30, 50, 100, and 200 μm samples (Figure S7) (PDF)

## References

[ref1] Fujishima A.; Honda A. TiO_2_ Photoelectrochemistry and Photocatalysis. Nature 1972, 238, 37–38. 10.1038/238037a0. 12635268

[ref2] Frank S. N.; Bard A. J. Heterogeneous Photocatalytic Oxidation of Cyanide Ion in Aqueous Solutions at Titanium Dioxide Powder. J. Am. Chem. Soc. 1977, 99, 303–304. 10.1021/ja00443a081.

[ref3] Ibhadon A. O.; Fitzpatrick P. Heterogeneous Photocatalysis: Recent Advances and Applications. Catalysts 2013, 3, 189–218. 10.3390/catal3010189.

[ref4] Dijkstra M. F. J.; Michorius A.; Buwalda H.; Panneman H. J.; Winkelman J. G. M.; Beenackers A. A. C. M. Comparison of the Efficiency of Immobilized and Suspended Systems in Photocatalytic Degradation. Catal. Today 2001, 66, 487–494. 10.1016/S0920-5861(01)00257-7.

[ref5] Bideau M.; Claudel B.; Dubien C.; Faure L.; Kazouan H. On the “Immobilization” of Titanium Dioxide in the Photocatalytic Oxidation of Spent Waters. J. Photochem. Photobiol., A 1995, 91, 137–144. 10.1016/1010-6030(95)04098-Z.

[ref6] Lin L. Y.; Yeh M. H.; Lee C. P.; Chen Y. H.; Vittal R.; Ho K. C. Metal-Based Flexible TiO_2_ Photoanode with Titanium Oxide Nanotubes as the Underlayer for Enhancement of Performance of a Dye-Sensitized Solar Cell. Electrochim. Acta 2011, 57, 270–276. 10.1016/j.electacta.2011.03.065.

[ref7] Farahani E.; Mohammadpour R. Fabrication of Flexible Self-Powered Humidity Sensor Based on Super-Hydrophilic Titanium Oxide Nanotube Arrays. Sci. Rep. 2020, 10, 13032 10.1038/s41598-020-70031-z. 32747666 PMC7400629

[ref8] Gulati K.; Santos A.; Findlay D.; Losic D. Optimizing Anodization Conditions for the Growth of Titania Nanotubes on Curved Surfaces. J. Phys. Chem. C 2015, 119, 16033–16045. 10.1021/acs.jpcc.5b03383.

[ref9] Ghosh J. P.; Achari G.; Langford C. H. Design and Evaluation of a UV LED Photocatalytic Reactor Using Anodized TiO_2_ Nanotubes. Water Environ. Res. 2016, 88, 785–791. 10.2175/106143015X14362865226879. 26488573

[ref10] Macák J. M.; Tsuchiya H.; Schmuki P. High-Aspect-Ratio TiO_2_ Nanotubes by Anodization of Titanium. Angew. Chem., Int. Ed. 2005, 44, 2100–2102. 10.1002/anie.200462459. 15736238

[ref11] Alivov Y.; Fan Z. Y.; Johnstone D. Titanium Nanotubes Grown by Titanium Anodization. J. Appl. Phys. 2009, 106, 034314 10.1063/1.3187927.

[ref12] Macak J. M.; Tsuchiya H.; Ghicov A.; Yasuda K.; Hahn R.; Bauer S.; Schmuki P. TiO_2_ Nanotubes: Self-Organized Electrochemical Formation, Properties and Applications. Curr. Opin. Solid State Mater. Sci. 2007, 11, 3–18. 10.1016/j.cossms.2007.08.004.

[ref13] Xie Z. B.; Blackwood D. J. Effects of Anodization Parameters on the Formation of Titania Nanotubes in Ethylene Glycol. Electrochim. Acta 2010, 56, 905–912. 10.1016/j.electacta.2010.10.004.

[ref14] Prakasam H. E.; Shankar K.; Paulose M.; Varghese O. K.; Grimes C. A. A New Benchmark for TiO_2_ Nanotube Array Growth by Anodization. J. Phys. Chem. C 2007, 111, 7235–7241. 10.1021/jp070273h.

[ref15] Macak J. M.; Schmuki P. Anodic Growth of Self-Organized Anodic TiO_2_ Nanotubes in Viscous Electrolytes. Electrochim. Acta 2006, 52, 1258–1264. 10.1016/j.electacta.2006.07.021.

[ref16] Ozkan S.; Mazare A.; Schmuki P. Critical Parameters and Factors in the Formation of Spaced TiO_2_ nanotubes by Self-Organizing Anodization. Electrochim. Acta 2018, 268, 435–447. 10.1016/j.electacta.2018.02.120.

[ref17] Omidvar H.; Goodarzi S.; Seif A.; Azadmehr A. R. Influence of Anodization Parameters on the Morphology of TiO_2_ nanotube Arrays. Superlattices Microstruct. 2011, 50, 26–39. 10.1016/j.spmi.2011.04.006.

[ref18] Roy P.; Berger S.; Schmuki P. TiO_2_ nanotubes: Synthesis and Applications. Angew. Chem., Int. Ed. 2011, 50, 2904–2939. 10.1002/anie.201001374. 21394857

[ref19] Quan X.; Yang S.; Ruan X.; Zhao H. Preparation of Titania Nanotubes and Their Environmental Applications as Electrode. Environ. Sci. Technol. 2005, 39, 3770–3775. 10.1021/es048684o. 15952384

[ref20] Schmidt-Stein F.; Thiemann S.; Berger S.; Hahn R.; Schmuki P. Mechanical Properties of Anatase and Semi-Metallic TiO_2_ Nanotubes. Acta Mater. 2010, 58, 6317–6323. 10.1016/j.actamat.2010.07.053.

[ref21] Macak J. M.; Zlamal M.; Krysa J.; Schmuki P. Self-Organized TiO_2_ Nanotube Layers as Highly Efficient Photocatalysts. Small 2007, 3, 300–304. 10.1002/smll.200600426. 17230591

[ref22] Ghicov A.; Schmuki P. Self-ordering Electrochemistry: A Review on Growth and Functionality of TiO_2_ Nanotubes and Other Self-Aligned MOx Structures. Chem. Commun. 2009, 20, 2791 10.1039/b822726h. 19436878

[ref23] Macak J. M.; Hildebrand H.; Marten-Jahns U.; Schmuki P. Mechanistic Aspects and Growth of Large Diameter Self-Organized TiO_2_ Nanotubes. J. Electroanal. Chem. 2008, 621, 254–266. 10.1016/j.jelechem.2008.01.005.

[ref24] Zhou X.; Liu N.; Schmuki P. Photocatalysis with TiO_2_ Nanotubes: “Colorful” Reactivity and Designing Site-Specific Photocatalytic Centers into TiO_2_ Nanotubes. ACS Catal. 2017, 7, 3210–3235. 10.1021/acscatal.6b03709.

[ref25] Krbal M.; Sopha H.; Pohl D.; Benes L.; Damm C.; Rellinghaus B.; Kupčík J.; Bezdička P.; Šubrt J.; Macak J. M. Self-Organized TiO_2_ Nanotubes Grown on Ti Substrates with Different Crystallographic Preferential Orientations: Local Structure of TiO_2_ Nanotubes vs. Photo-Electrochemical Response. Electrochim. Acta 2018, 264, 393–399. 10.1016/j.electacta.2018.01.113.

[ref26] Macak J. M.; Jarosova M.; Jäger A.; Sopha H.; Klementová M. Influence of the Ti Microstructure on Anodic Self-Organized TiO_2_ Nanotube Layers Produced in Ethylene Glycol Electrolytes. Appl. Surf. Sci. 2016, 371, 607–612. 10.1016/j.apsusc.2016.03.012.

[ref27] Leonardi S.; Li Bassi A.; Russo V.; Di Fonzo F.; Paschos O.; Murray T. M.; Efstathiadis H.; Kunze J. TiO_2_ Nanotubes: Interdependence of Substrate Grain Orientation and Growth Characteristics. J. Phys. Chem. C 2012, 116, 384–392. 10.1021/jp209418n.

[ref28] Leonardi S.; Russo V.; Li Bassi A.; Di Fonzo F.; Murray T. M.; Efstathiadis H.; Agnoli A.; Kunze-Liebhäuser J. TiO_2_ Nanotubes: Interdependence of Substrate Grain Orientation and Growth Rate. ACS Appl. Mater. Interfaces 2015, 7, 1662–1668. 10.1021/am507181p. 25545715

[ref29] Sopha H.; Tesar K.; Knotek P.; Jäger A.; Hromadko L.; Macak J. M. TiO_2_ Nanotubes Grown on Ti Substrates with Different Microstructure. Mater. Res. Bull. 2018, 103, 197–204. 10.1016/j.materresbull.2018.03.036.

[ref30] Hyam R. S.; Choi D. Effects of Titanium Foil Thickness on TiO_2_ Nanostructures Synthesized by Anodization. RSC Adv. 2013, 3, 7057–7063. 10.1039/c3ra40581h.

[ref31] Utsunomiya H.; Abe K.; Matsumoto R. Formation of Roll Coating in Cold Rolling of Titanium Sheets. Procedia Eng. 2017, 207, 1367–1372. 10.1016/j.proeng.2017.10.898.

[ref32] Utsunomiya H.; Kameyama S.; Matsumoto R. Contact Resistance between Roll and Titanium Sheet during Cold Rolling. CIRP Ann. 2019, 68, 305–308. 10.1016/j.cirp.2019.04.032.

[ref33] Wang S.; Niu L.; Chen C.; Pang Y.; Liao B.; Zhong Z. H.; Lu P.; Li P.; Wu X. D.; Coenen J. W.; Cao L. F.; Wu Y. C. Size Effects on the Tensile Properties and Deformation Mechanism of Commercial Pure Titanium Foils. Mater. Sci. Eng., A 2018, 730, 244–261. 10.1016/j.msea.2018.06.009.

[ref34] Ghosh A.; Singh A.; Gurao N. P. Effect of Rolling Mode and Annealing Temperature on Microstructure and Texture of Commercially Pure-Titanium. Mater. Charact. 2017, 125, 83–93. 10.1016/j.matchar.2017.01.022.

[ref35] Chun Y. B.; Yu S. H.; Semiatin S. L.; Hwang S. K. Effect of Deformation Twinning on Microstructure and Texture Evolution during Cold Rolling of CP-Titanium. Mater. Sci. Eng., A 2005, 398, 209–219. 10.1016/j.msea.2005.03.019.

[ref36] Liu N.; Wang Y.; He W.-j.; Li J.; Chapuis A.; Luan B.-f.; Liu Q. Microstructure and Textural Evolution during Cold Rolling and Annealing of Commercially Pure Titanium Sheet. Trans. Nonferrous Met. Soc. China 2018, 28, 1123–1131. 10.1016/S1003-6326(18)64748-X.

[ref37] Zherebtsov S. V.; Dyakonov G. S.; Salem A. A.; Malysheva S. P.; Salishchev G. A.; Semiatin S. L. Evolution of Grain and Subgrain Structure during Cold Rolling of Commercial-Purity Titanium. Mater. Sci. Eng., A 2011, 528, 3474–3479. 10.1016/j.msea.2011.01.039.

[ref38] Hu N.; Gao N.; Starink M. J. The Influence of Surface Roughness and High Pressure Torsion on the Growth of Anodic Titania Nanotubes on Pure Titanium. Appl. Surf. Sci. 2016, 387, 1010–1020. 10.1016/j.apsusc.2016.07.036.

[ref39] Lu K.; Tian Z.; Geldmeier J. A. Polishing Effect on Anodic Titania Nanotube Formation. Electrochim. Acta 2011, 56, 6014–6020. 10.1016/j.electacta.2011.04.098.

[ref40] Sopha H.; Jäger A.; Knotek P.; Tesař K.; Jarosova M.; Macak J. M. Self-Organized Anodic TiO_2_ Nanotube Layers: Influence of the Ti Substrate on Nanotube Growth and Dimensions. Electrochim. Acta 2016, 190, 744–752. 10.1016/j.electacta.2015.12.121.

[ref41] Wang X.; Li Y.; Song H.; Huang Y.; Su R.; Besenbacher F. Fluoride Concentration Controlled TiO_2_ Nanotubes: The Interplay of Microstructure and Photocatalytic Performance. RSC Adv. 2016, 6, 18333–18339. 10.1039/C5RA24732B.

[ref42] Fraoucene H.; Sugiawati V. A.; Hatem D.; Belkaid M. S.; Vacandio F.; Eyraud M.; Pasquinelli M.; Djenizian T. Optical and Electrochemical Properties of Self-Organized TiO_2_ Nanotube Arrays From Anodized Ti–6Al–4V Alloy. Front. Chem. 2019, 7, 66 10.3389/fchem.2019.00066. 30800655 PMC6375903

[ref43] Wang Y. N.; Huang J. C. Texture Analysis in Hexagonal Materials. Mater. Chem. Phys. 2003, 81, 11–26. 10.1016/S0254-0584(03)00168-8.

[ref44] Zhong Y.; Yin F.; Nagai K. Role of Deformation Twin on Texture Evolution in Cold-Rolled Commercial-Purity Ti. J. Mater. Res. 2008, 23, 2954–2966. 10.1557/JMR.2008.0354.

[ref45] Salishchev G. A.; Mironov S. Y. Effect of Grain Size on Mechanical Properties of Commercially Pure Titanium. Russ. Phys. J. 2001, 44, 596–601. 10.1023/A:1012539711156.

[ref46] Liu N.; Mirabolghasemi H.; Lee K.; Albu S. P.; Tighineanu A.; Altomare M.; Schmuki P. Anodic TiO_2_ Nanotubes: Double Walled vs. Single Walled. Faraday Discuss. 2013, 164, 107–116. 10.1039/c3fd00020f. 24466660

[ref47] Albu S. P.; Ghicov A.; Aldabergenova S.; Drechsel P.; LeClere D.; Thompson G. E.; Macak J. M.; Schmuki P. Formation of Double-Walled TiO_2_ Nanotubes and Robust Anatase Membranes. Adv. Mater. 2008, 20, 4135–4139. 10.1002/adma.200801189.

[ref48] Berger S.; Albu S. P.; Schmidt-Stein F.; Hildebrand H.; Schmuki P.; Hammond J. S.; Paul D. F.; Reichlmaier S. The Origin for Tubular Growth of TiO_2_ Nanotubes: A Fluoride Rich Layer between Tube-Walls. Surf. Sci. 2011, 605, L57–L60. 10.1016/j.susc.2011.06.019.

[ref49] ASTM International Standard Test Methods for Measuring Adhesion by Tape Test D3359-09, 1–8.

[ref50] Mittal K. L. Adhesion Measurement of Thin Films. Electrocomponent Sci. Technol. 1976, 3, 21–42. 10.1155/APEC.3.21.

[ref51] Cao S.; Huang W.; Wu L.; Tian M.; Song Y. On the Interfacial Adhesion between TiO_2_ Nanotube Array Layer and Ti Substrate. Langmuir 2018, 34, 13888–13896. 10.1021/acs.langmuir.8b03408. 30362766

[ref52] Zou J. P.; Wang R. Z. Crack Initiation, Propagation and Saturation of TiO_2_ Nanotube Film. Trans. Nonferrous Met. Soc. China 2012, 22, 627–633. 10.1016/S1003-6326(11)61224-7.

[ref53] Luo J.; Li B.; Ajami S.; Ma S.; Zhou F.; Liu C. Growth of TiO_2_ Nanotube on Titanium Substrate to Enhance Its Biotribological Performance and Biocorrosion Resistance. J. Bionic Eng. 2019, 16, 1039–1051. 10.1007/s42235-019-0116-2.

[ref54] Muangmora R.; Kemacheevakul P.; Punyapalakul P.; Chuangchote S. Enhanced Photocatalytic Degradation of Caffeine Using Titanium Dioxide Photocatalyst Immobilized on Circular Glass Sheets under Ultraviolet C Irradiation. Catalysts 2020, 10, 964 10.3390/catal10090964.

[ref55] Lorenzetti M.; Biglino D.; Novaka S.; Kobe S. Photoinduced Properties of Nanocrystalline TiO_2_-Anatase Coating on Ti-Based Bone Implants. Mater. Sci. Eng., C 2014, 37, 390–398. 10.1016/j.msec.2014.01.029. 24582265

[ref56] Adán C.; Marugán J.; Sánchez E.; Pablos C.; Van Grieken R. Understanding the Effect of Morphology on the Photocatalytic Activity of TiO_2_ Nanotube Array Electrodes. Electrochim. Acta 2016, 191, 521–529. 10.1016/j.electacta.2016.01.088.

[ref57] Ma L.; Zhang Q.; Zhao Q.; Li Z.; Ji C.; Bu H.; Xu X. J. Fabrication and Photoluminescence Properties of Ridged TiO_2_ Nanotube Arrays. J. Mater. Sci. Mater. Electron. 2014, 25, 3290–3294. 10.1007/s10854-014-2016-x.

[ref58] Lei Y.; Zhang L. D.; Meng G. W.; Li G. H.; Zhang X. Y.; Liang C. H.; Chen W.; Wang S. X. Preparation and Photoluminescence of Highly Ordered TiO_2_ Nanowire Arrays. Appl. Phys. Lett. 2001, 78, 1125–1127. 10.1063/1.1350959.

[ref59] Etacheri V.; Di C.; Schneider J.; Bahnemann D.; Pillai S. C. Visible-Light Activation of TiO_2_ Photocatalysts: Advances in Theory and Experiments. J. Photochem. Photobiol., C 2015, 25, 1–29. 10.1016/j.jphotochemrev.2015.08.003.

[ref60] Pan X.; Yang M. Q.; Fu X.; Zhang N.; Xu Y. J. Defective TiO_2_ with Oxygen Vacancies: Synthesis, Properties and Photocatalytic Applications. Nanoscale 2013, 5, 3601–3614. 10.1039/c3nr00476g. 23532413

[ref61] Taieb S. B.; Assaker I. B.; Bardaoui A.; Gannouni M.; Souissi A.; Nowak S.; Mouton L.; Ammar S.; Chtourou R. Correlation between Titanium Foil Substrate Purity and TiO_2_ NTs; Physical and Electrochemical Properties for Enhanced Photoelectrochemical Applications. Int. J. Hydrogen Energy 2016, 41, 6230–6239. 10.1016/j.ijhydene.2016.03.043.

[ref62] Ferreira A. P. Caffeine as an Environmental Indicator for Assessing Urban Aquatic Ecosystems. Cad. Saude Publica 2005, 21, 1884–1892. 10.1590/S0102-311X2005000600038. 16410875

[ref63] Ahmad Bhawani S.; Fong S. S.; Mohamad Ibrahim M. N. Spectrophotometric Analysis of Caffeine. Int. J. Anal. Chem. 2015, 2015, 1–7. 10.1155/2015/170239. PMC4641934 26604926

