# Supplementary material for: Toward a Flexible and Efficient TiO2 Photocatalyst Immobilized on a Titanium Foil
Source: ACS Omega. 2021 Aug 25;6(36):23233–42. doi: 10.1021/acsomega.1c02862 (PMC8444206; doi:10.1021/acsomega.1c02862)
Supplement: Supplementary file 1 — ao1c02862_si_001.pdf [file ao1c02862_si_001.pdf]

Supporting Information

Toward a Flexible and Efficient TiO_2 Photocatalyst Immobilized on a Titanium Foil

Živa Marinko*[,†,‡], Luka Suhadolnik[†], Barbara Šetina Batič[§], Vid Simon Šelih[‖], Boris Majaron[⊥,#], Janez Kovač[∇], and Miran Čeh[†]

[†]Department for Nanostructured Materials, Jožef Stefan Institute, Jamova 39, 1000 Ljubljana, Slovenia; luka.suhadolnik@ijs.si (L.S.); miran.ceh@ijs.si (M.Č.)

[‡]Jozef Stefan International Postgraduate School, Jamova 39, 1000 Ljubljana, Slovenia

[§]Vacuum Science and Optoelectronics, Institute of Metals and Technology, Lepi pot 11, 1000 Ljubljana, Slovenia; barbara.setina@imt.si (B.Š.)

[‖]Center for Validation Tehnologies and Analytics & Department of Analytical Chemistry, National Institute of Chemistry, Hajdrihova 19, 1001 Ljubljana, Slovenia; vid.selih@ki.si (V.S.Š.)

[⊥]Department of Complex Matter, Jožef Stefan Institute, Jamova 39, 1000 Ljubljana, Slovenia; boris.majaron@ijs.si (B.M.)

[#]Faculty of Mathematics and Physics, University of Ljubljana, Jadranska 19, 1000 Ljubljana, Slovenia (B.M.)

[∇]Department of Surface Engineering, Jožef Stefan Institute, Jamova 39, 1000 Ljubljana, Slovenia; janez.kovac@ijs.si (J.K)

*Email: ziva.marinko@ijs.si (Ž.M.). Tel: +386-1-4773-931

Keywords

TiO_2 nanotubes; anodic oxidation; flexible photocatalyst; metal titanium foil

S1. Microstructure Properties of the Starting Titanium Foils

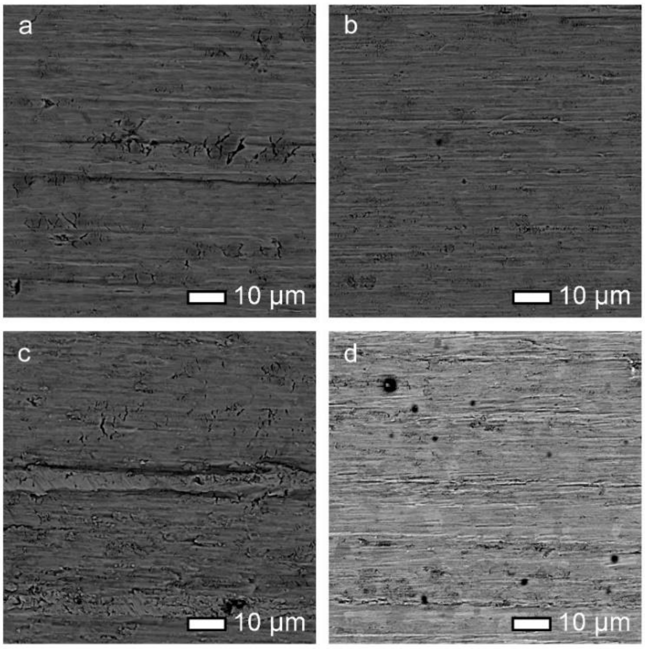

Figure S1. SEM images of metal titanium foil of different thicknesses. (a) 30 μm, (b) 50 μm, (c) 100 μm, (d) 200 μm.

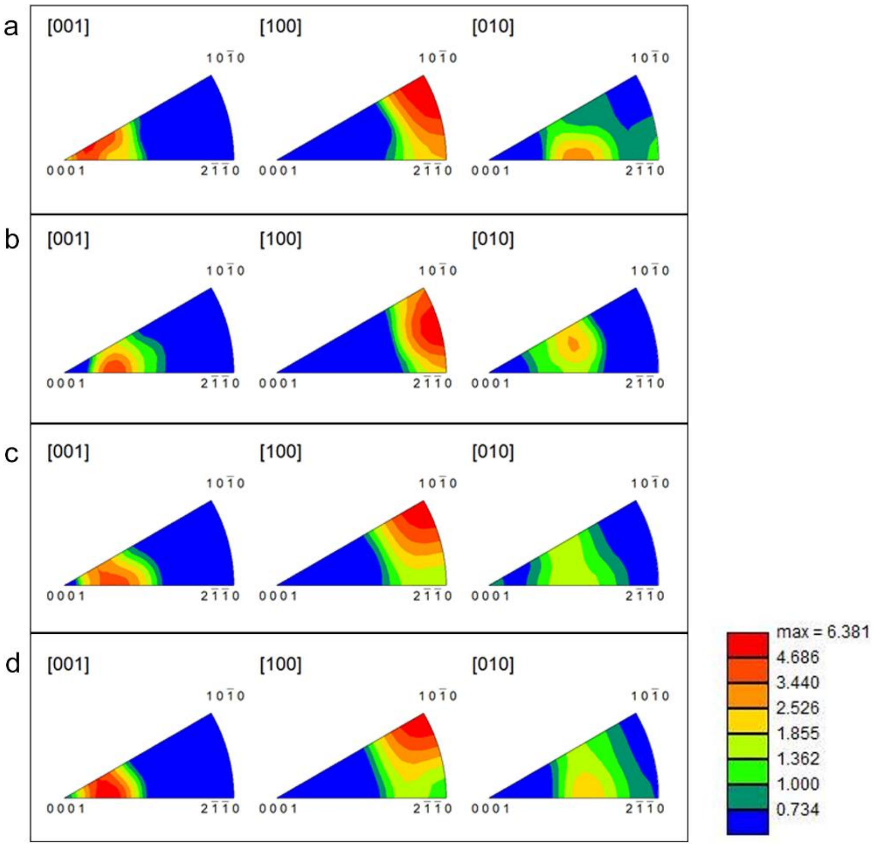

Figure S2. Inverse pole figures (IPFs) for all samples. (a) 30 μm, (b) 50 μm, (c) 100 μm, (d) 200 μm.

S2. Characteristics of the Annealed TiO_2 NT Layers

XPS analyses of all the samples were performed. The results showed that the surface's chemical composition is very comparable among the samples (Table S1). Additionally, the high-energy resolution XPS spectra Ti 2p, O 1s and C 1s are presented in Figure S3. The spectra of Ti 2p (a) from all samples are very similar. The Ti $2p_{3/2}$ peak at 458.6 eV can be assigned to Ti(4+) in TiO_2 oxide. O 1s spectra (b) are also very similar among the samples. The spectra show a large peak at 530.0 eV, which is assigned to O(2-) in the TiO_2 oxide lattice, and a small peak at 531.6 eV, which is about 15% of the total O 1s spectra and can be assigned to the OH or O vacancies in the TiO_2 oxide layer. C 1s spectra (c) are also very similar for all the samples and probably originate from the contamination or electrolyte content. Additionally, traces of nitrogen as N 1s and potassium as K 2p peaks were detected.

Table S1. Surface chemical composition in at.% and the O/Ti ratio for all samples.

Sample	Ti	O	C	O/Ti
30 µm	21.1	52.5	26.4	2.49
50 µm	20.1	47.7	32.3	2.37
100 µm	22.6	53.7	23.7	2.38
200 µm	22.7	54.1	23.2	2.38

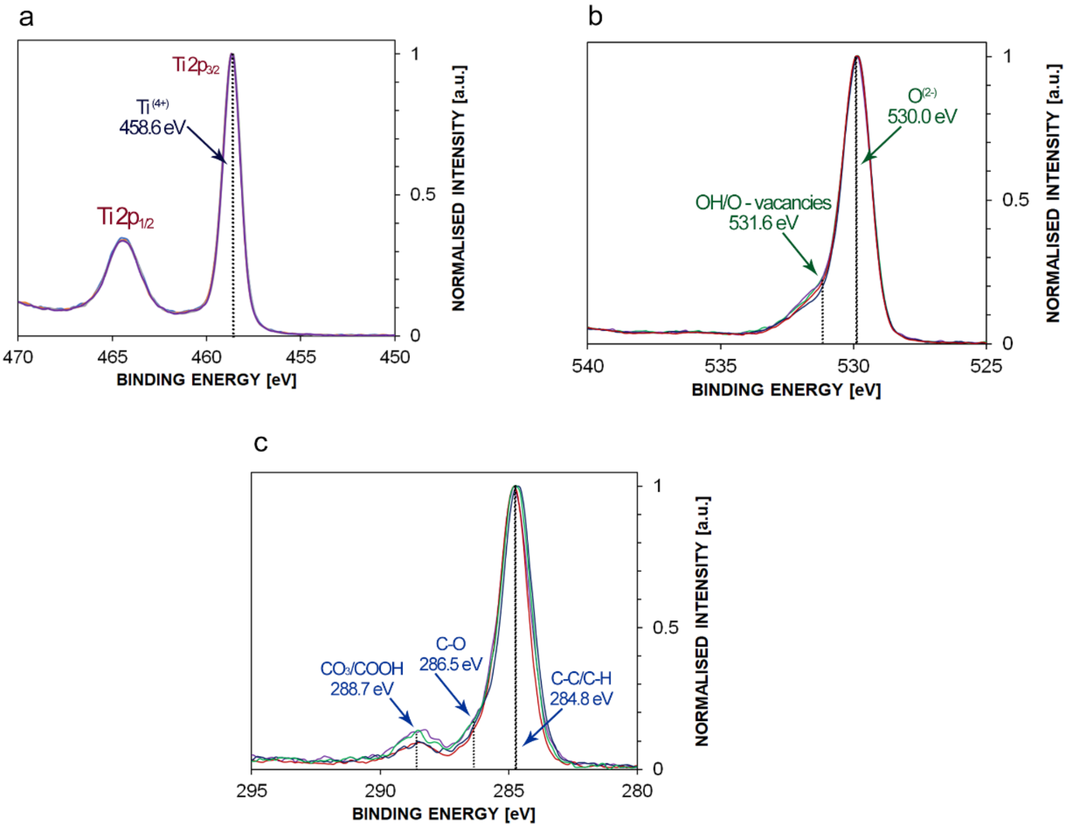

Figure S3. High-resolution XPS spectra for (a) Ti 2p, (b) O 1s and, (c) C 1s peaks for all TiO_2 NT samples.

The TiO_x NT layers grown by anodic oxidation are amorphous. Annealing at 450 °C for 1 hour transforms the amorphous NTs into the anatase phase. XRD analyses performed on all four samples

confirmed the phase transformation. At the same time, annealing significantly improves the contact between the titanium foil and the nanotubes.[1] All diffractograms in Figure S4 show distinct peaks related to the anatase phase at 2θ angles. The diffraction peaks corresponding to the titanium foil can be observed in each XRD spectra due to the relatively thin and porous TiO_2 NT layers.

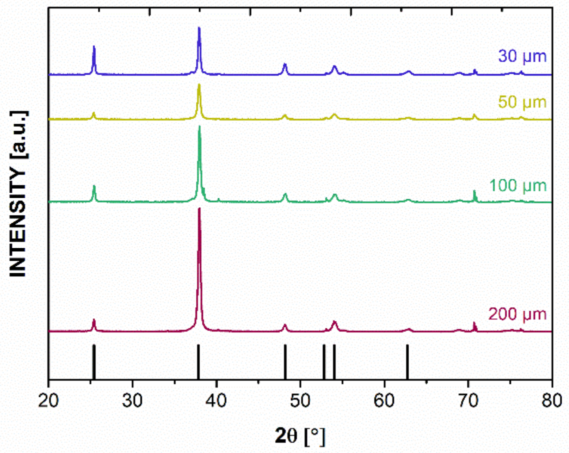

Figure S4. XRD measurements for each sample. The diffraction peaks corresponding to the titanium foil are not marked.

Analyses of the TiO_2 NTs' top surface, bottom surface, and cross-section were carried out with FEG-SEM. They show significant differences between the samples (Figure S5). Many wide, short cracks were observed on the thinnest sample's surface in the transverse and longitudinal directions. The top oxide layer was thin and, in some areas, absent. At those areas, the NT surface was etched. On the 50-µm sample, the density of the cracks decreased; they were shorter and transverse-oriented. The top oxide layer is the thinnest; NTs are seen more clearly. In contrast, was the 100 µm sample surface. Very few cracks can be observed. The TiO_2 NTs' surface is uneven with sponge-like areas of etched NTs and regularly looked like NTs with a thick oxide layer on top. Finally, the thickest foil, i.e., 200 µm, had the most even surface with the thinnest top oxide layer. Although the cracks were long and wide, they were rare, with the NTs closely packed, and the boundary between the individual NTs clearly seen.

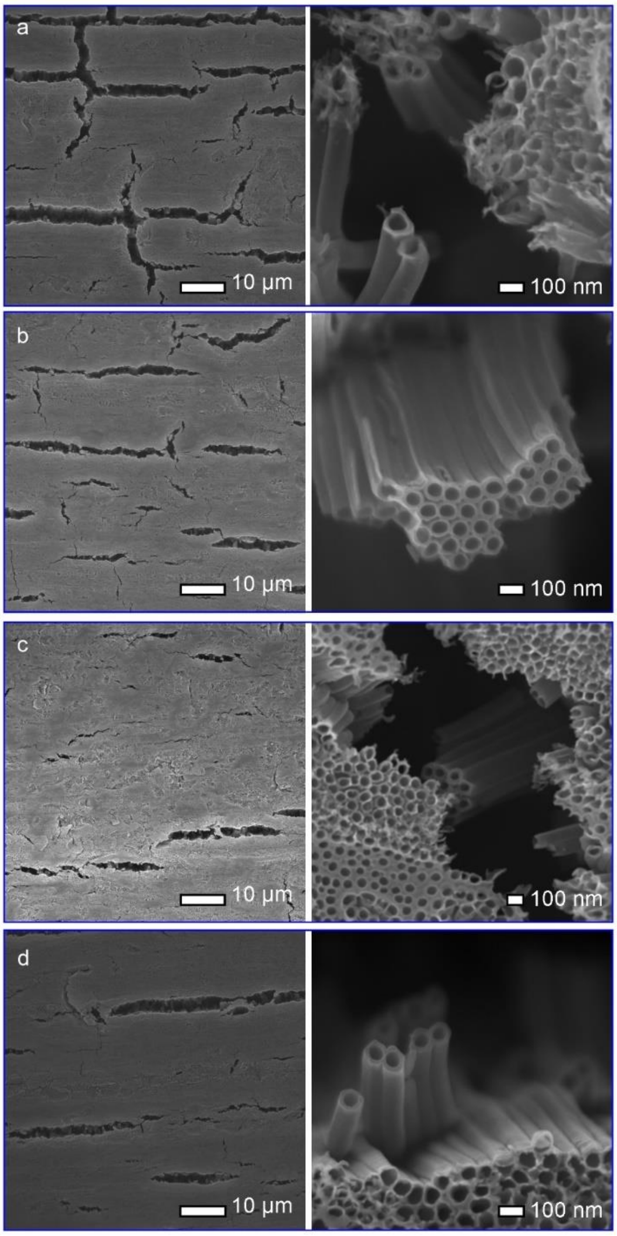

Figure S5. FEG-SEM micrographs of TiO_2 NTs for each foil thickness, (a) 30 μm, (b) 50 μm, (c) 100 μm, (d) 200 μm samples.

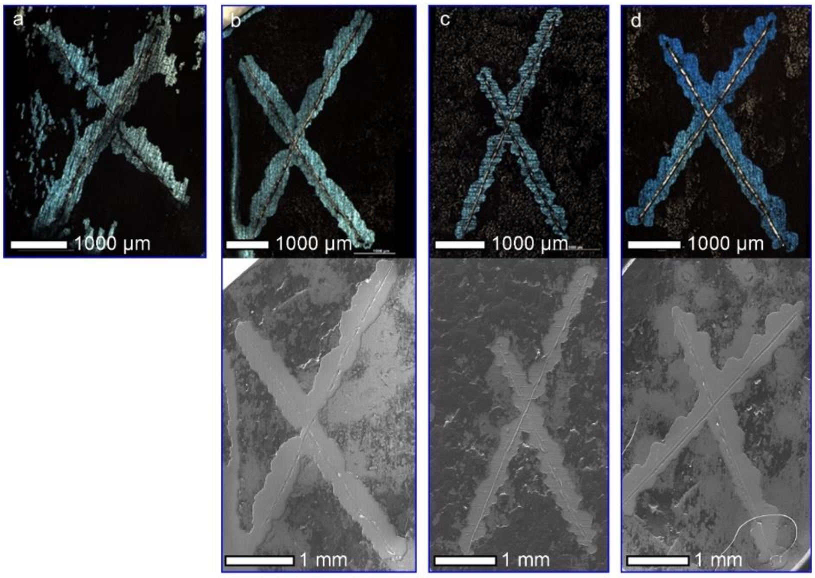

Figure S6. Adhesion of TiO_2 NTs performed with Scotch-tape test on (a) 30 µm, (b) 50 µm, (c) 100 µm, (d) 200 µm samples. Black areas on the upper micrographs are the TiO_2 NTs' remains, and blue areas are oxidised titanium foils from which the TiO_2 NTs were removed due to weak adhesion. Below are micrographs taken on FEG-SEM. Adhesion of 30 µm sample NTs was poor and did not withstand the preparation for FEG-SEM observation.

S3. Measurements of Photocatalytic Degradation

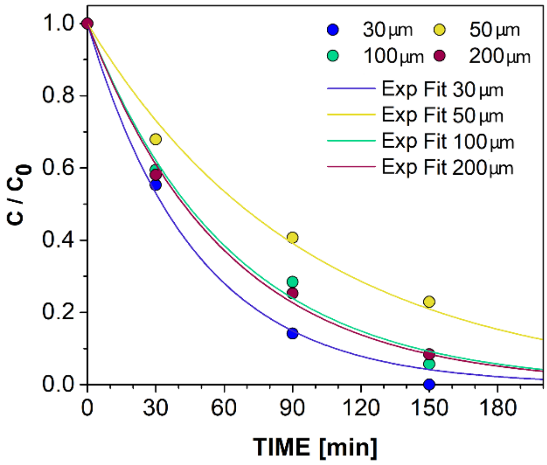

Figure S7. Photocatalytic degradation of caffeine (dots) for all four samples; and exponential curve fitting (lines) for 30, 50, 100, and 200 µm samples.

REFERENCES

(1) Xiong, J.; Wang, X.; Li, Y.; Hodgson, P. D. Interfacial Chemistry and Adhesion between Titanium Dioxide Nanotube Layers and Titanium Substrates. *J. Phys. Chem. C* **2011**, *115*, 4768–4772.
